# Trust and Health Information Exchanges: Qualitative Analysis of the Intent to Share Personal Health Information

**DOI:** 10.2196/41635

**Published:** 2023-08-30

**Authors:** Julia Busch-Casler, Marija Radic

**Affiliations:** 1 Fraunhofer Center for International Management and Knowledge Economy IMW Leipzig Germany

**Keywords:** trust, eHealth, data sharing, sharing personal health information, privacy, security, health information exchange, consent, data exchange, belief-attitude-intention, behavior formation

## Abstract

**Background:**

Digital health has the potential to improve the quality of care, reduce health care costs, and increase patient satisfaction. Patient acceptance and consent are a prerequisite for effective sharing of personal health information (PHI) through health information exchanges (HIEs). Patients need to form and retain trust in the system(s) they use to leverage the full potential of digital health. Germany is at the forefront of approving digital treatment options with cost coverage through statutory health insurance. However, the German population has a high level of technology skepticism and a low level of trust, providing a good basis to illuminate various facets of eHealth trust formation.

**Objective:**

In a German setting, we aimed to answer the question, How does an individual form a behavioral intent to share PHI with an HIE platform? We discussed trust and informed consent through (1) synthesizing the main influence factor models into a complex model of trust in HIE, (2) providing initial validation of influence factors based on a qualitative study with patient interviews, and (3) developing a model of trust formation for digital health apps.

**Methods:**

We developed a complex model of the formation of trust and the intent to share PHI. We provided initial validation of the influence factors through 20 qualitative, semistructured interviews in the German health care setting and used a deductive coding approach to analyze the data.

**Results:**

We found that German patients show a positive intent to share their PHI with HIEs under certain conditions. These include (perceived) information security and a noncommercial organization as the recipient of the PHI. Technology experience, age, policy and regulation, and a disposition to trust play an important role in an individual’s privacy concern, which, combined with social influence, affects trust formation on a cognitive and emotional level. We found a high level of cognitive trust in health care and noncommercial research institutions but distrust in commercial entities. We further found that in-person interactions with physicians increase trust in digital health apps and PHI sharing. Patients’ emotional trust depends on disposition and social influences. To form their intent to share, patients undergo a privacy calculus. Hereby, the individual’s benefit (eg, convenience), benefits for the individual’s own health, and the benefits for public welfare often outweigh the perceived risks of sharing PHI.

**Conclusions:**

With the higher demand for timely PHI, HIE providers will need to clearly communicate the benefits of their solutions and their information security measures to health care providers (physicians, nursing and administrative staff) and patients and include them as key partners to increase trust. Offering easy access and educational measures as well as the option for specific consent may increase patients’ trust and their intention to share PHI.

## Introduction

### Background

Data-driven medicine promises better care and more efficient health care processes. Digital health information exchanges (HIEs), electronic health records (EHRs), and eHealth and mobile health (mHealth) apps have become increasingly relevant for sharing personal health information (PHI) in the past years. Countries aim to adopt and implement HIEs to improve the quality of care, reduce health care costs, und increase patient outcomes and satisfaction [1]. Germany is no exception. In 2019, Germany passed a law approving the prescription of mHealth apps by doctors whereby the costs are covered by the German statutory health insurance. All insured people are eligible to use registered mHealth apps as part of standard care [2]. However, uptake has been slow because of restraints from both patients and providers [3,4]. A recent study among German citizens [5] found that almost 25% of the respondents believe that technology creates more problems than it solves, thus indicating that Germans are highly skeptical toward technology overall. This is in line with prior research on country-specific trust levels [6,7], where Germany is associated with rather low levels of trust compared to other countries.

Patient acceptance and opt-in are crucial for efficient use of HIEs (we subsume EHRs, mHealth apps, and eHealth apps under the term “HIEs” for purposes of readability). Patients need to trust that the information security measures and privacy policies of the HIE provider are sufficient to protect their PHI [8,9]. Providers must explain these policies to the patient and show that they are upheld. Several studies have found that most patients have a positive attitude toward EHRs for reasons of convenience, completeness, and ease of communication [4,10-13]. However, PHI is considered highly sensitive. Data breaches can potentially have significant negative consequences for the patients involved [14]. Patients, although excited about the possibilities of EHRs [10,15-17], might not fully understand the impact their sharing decisions may have. They may even be reluctant to share their PHI digitally after witnessing data breaches [18]. Privacy concerns are the largest barrier to sharing PHI [16,19]. Trust in the safety and soundness of technological solutions has a strong impact on user opt-in [9,19-21]. Backhaus [22] described the trust of a user in a technical system as the expectation that the system will perform certain tasks based on the user’s wishes and assumptions without misusing their vulnerability caused by the execution of the process. Trust in digital health apps is strongly linked to trust in the respective health care provider [23]. Buhr et al [23], for example, found that Germans trust governmental institutions, such as the statutory health insurance, more than private institutions. Dhopeshwarkar et al [20] found that patients trust physicians regarding accessing health care files. Considering these developments, patients need to become the sovereign of their own PHI [24]. They need to be able to provide informed consent on what should be shared through HIEs and who can use PHI stored in their EHRs.

There have been multiple calls for more research on the subject, followed by an upswing in recent years [25]. Looking at the specific case of Germany in the context of regulatory initiatives [26] and a comparatively low trust level [27], however, may provide additional insight into patients’ behavioral intentions [28,29] and measures that HIE providers can undertake to increase the level of trust in their solutions and processes. Our research aimed to answer the following research question: How does an individual form a behavioral intent to share PHI with an HIE platform? We contributed to the discussion of trust and informed consent in digital health in the following ways: (1) We derived a complex model of trust in an eHealth app and intent formation to share PHI based on the belief-attitude-intention framework, (2) provided initial exploratory validation of influence factors through a qualitative analysis process with interviews of German patients, and (3) developed a model of trust formation for eHealth apps.

### Initial Model

Trust in and acceptance of eHealth apps have become a more prevalent research area in recent years due to the increasing uptake of HIEs and the rise in virtual interactions in the COVID-19 pandemic years [23,25,28]. Different approaches try to assess trust in and user acceptance of (health) information technology and the sharing of PHI. Consumer acceptance and use of technology is often assessed based on the Unified Theory of Acceptance and Use of Technology (UTAUT) and its extensions and adaptations [4,30,31]. The model has been applied to the health care context [4,12,13,21] and has also been enhanced with health behavior theories, such as the Health Belief Model, protection motivation theory, and social cognitive theory [24]. Abdelhamid [31], for example, adapted the UTAUT model to PHI sharing with HIEs and used privacy concerns, social influence, trust in health care professionals, health concerns, and perceived usefulness as the main variables for his quantitative study. He found that all factors except for privacy concerns have a positive impact on the sharing intention. More customized sharing choices may mitigate the negative effect of privacy concerns on PHI-sharing intention.

Privacy concerns are often stated as the main barrier to sharing of PHI. They are, however, not always part of the (adapted) Extended Unified Theory of Acceptance and Use of Technology (UTAUT2) models. The Antecedent-Privacy Concern-Outcome (APCO) model presents 1 approach for the analysis of privacy concerns [32-35]. Shen et al [35], for example, developed an eHealth trust model based on the APCO approach and suggested personality, tech-savviness, eHealth awareness, health care perception, privacy experience, demographics, and culture as antecedents of privacy concerns. The authors described trusting belief as well as policy and regulation as moderating factors. In the outcome stage, they distinguished between a privacy calculus and the final behavioral reaction.

Privacy concerns are often associated with the privacy calculus model [16,35-38]. Abdelhamid et al [16], for example, presented a model with the following variables: patient activation, issue involvement, privacy concerns, trust in providers, and patient-physician relationship. They found that privacy concerns negatively affect the intention to share PHI. This can only partially be mitigated by the other variables.

Trust is another factor in assessing the acceptance of sharing PHI, which is sometimes covered in the UTAUT2 adaptions [21] but also analyzed separately [39]. Trust is often distinguished into a personal disposition, cognitive trust (in systems, people, etc) and emotional trust [19,39]. Esmaeilzadeh [8], for example, examined the acceptance of HIEs using a complex model of trust formation. The main variables analyzed included trust in health care providers and perceived transparency of the HIE privacy policy, leading to cognitive trust in, first, the integrity of the HIEs and, second, the competency of the HIEs. The latter factors influence emotional trust, which then translates into opt-in and willingness to disclose information.

Given the multifaceted nature of the concepts presented, in this study, we aimed to integrate the previous findings into a comprehensive model.

## Methods

### Scientific Basis of the Initial Model

**Figure 1:**
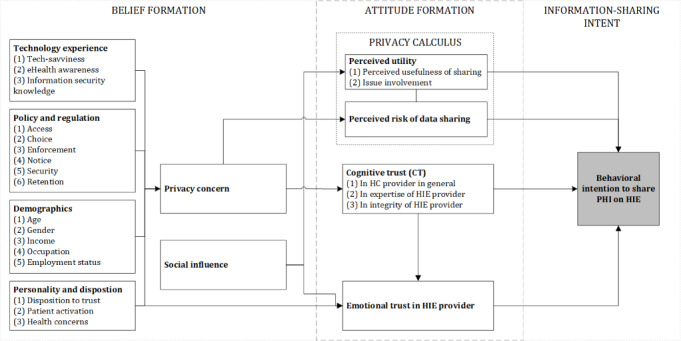
Conceptual model of eHealth trust formation and prevalidation (own illustration). HC: health care; HIE: health information exchange; PHI: personal health information.

Our initial model is depicted in Figure 1. An overall definition of the constructs used in the initial model can be found in Multimedia Appendix 1. We transferred the common belief-attitude-intention framework based on the theory of reasoned action [8,40] to a PHI-sharing setting. We divided our model into 3 stages: (1) belief formation, (2) attitude formation, and (3) information-sharing intent. We defined *privacy concern* as a belief referring to “the information the individual has about the object,” in this case the HIE [41]. We defined *trust* as an attitude, that is, the “favorable or unfavorable evaluation of an attitude object” [41]. We defined *intent* as “the subjective probability that the person will behave in a particular way vis-à-vis the attitude object” [41]. Beliefs are formed based on preconditions and previous experiences [40,41], which are sometimes referred to as values [41] or antecedents [32,35]. The antecedents of privacy concerns are depicted in the APCO model [32,35,42]. We added the antecedent component to the belief-attitude-intention framework to enhance the understanding of the belief formation. Behavioral intentions are influenced not only by trust but also by an individual’s *privacy calculus* [32,36,37], defined as the “cost-benefit analysis” of disclosing information [32,43]. We defined *privacy calculus* as an attitude, following the attitude definition of Stone et al [41].

#### Stage 1: Belief Formation

In the *belief formation stage*, an individual forms a belief about privacy and related risks of sharing PHI (privacy concern) based on antecedents. An individual has certain previous experiences with sharing information and (eHealth) technology, which we subsumed under *technology experience* [4]. We included an individual’s general experience with technology in the variable *tech-savviness* [35]. Since eHealth is a comparatively new topic for most individuals, we can only assess the awareness of eHealth of an individual [35]. We synthesized Shen et al’s [35] *privacy perspective* and related findings of Abdelhamid [31] and Hassandoust et al [37] into the item *information security knowledge* to cover potential experiences with data breaches and measures taken to protect an individual’s PHI in the light of the discussions of data sovereignty. We followed the following definition of information security: “Information security is the protection of information from a wide range of threats. This is achieved by managing a suitable set of security controls, policies and procedures within an Information Security Management System. The goal of general InfoSec is the ‘preservation of confidentiality, integrity and availability of information’ and includes such terms as the accountability of users, authentication, non-repudiation and reliability” [44]. Esmailzadeh [8] and Shen et al [35] have shown that an individual’s perception of policy and regulation influences their privacy beliefs. We included demographic factors as antecedents because studies have shown clear differences between the privacy beliefs of diverse demographic groups [4,5,45,46]. Finally, an individual has personality traits and dispositions, particularly a disposition to trust, that influence all interactions with the individual’s environment [19,47,48], including privacy concerns, attitudes, and the intent to share or withhold PHI [32,49]. In the eHealth setting, we argued in line with Abdelhamid et al [16,31] and added *health concerns* and *patient activation* into the initial dispositions. All the aforementioned factors lead to the formation of privacy concerns related to sharing an individual’s PHI. Individuals continuously interact in their specific social environment. Studies [31,37,50] have shown that social influence affects the intent to share via trust formation. We included *social influence* as an additional antecedent to trust and the privacy calculus, influencing the perceived utility of sharing PHI [31,37], and the emotional trust of an individual in an HIE.

#### Stage 2: Attitude Formation

In the *attitude formation stage*, an individual forms attitudes toward sharing PHI. These attitudes can be divided into the privacy calculus (see the previous section) and trust. The concept of trust has a composite definition [49,51,52]. The thoughts and decisions of an individual include both cognition and emotion [52], leading to a distinction between cognitive trust and emotional trust [52,53]. Cognitive trust in eHealth has different dimensions: First, patients develop a level of cognitive trust in their health care providers, which is necessary for the initial treatment. Based on trust transfer theory, individuals may transfer this established trust to the HIE [23,49,54]. To form a sharing intent through an HIE, however, individuals not only need to trust the health care institution but also have thoughts about the expertise and integrity of the HIE provider [8]. This is often not directly associated with the health care provider. Research shows that an individual’s privacy concern influences the risk associated with sharing PHI and well as the cognitive judgment whether to trust an entity in a digital setting [32,37]. The privacy calculus assesses the perceived utility of the sharing decision and compares it to the perceived risks associated with this decision [32,37]. Individuals value sharing data if they have a perceived benefit. In the case of PHI, patients may, for example, experience better or faster treatment. They may perceive a benefit because a certain health topic has personal relevance due to a particular health concern, which we captured as *issue involvement* [31]. The perceived risk refers to the loss or misuse of PHI because of, for example, data breaches and the associated perceived damages the individual incurs because of the data incident [35].

#### Stage 3: Information-Sharing Intent

Finally, in the *information-sharing intent stage*, the individual forms a behavioral intent to share PHI with the HIE. Contrary to Esmaeilzadeh [8,53], we did not differentiate between the opt-in intention and the willingness-to-share intention. Patients often do not actually have an option to opt in or out of an HIE [8], but rather, they have choices on what to share with an HIE. We regarded the willingness to share information with an HIE as the outcome of the trust formation model. We defined the willingness to share (health) information as the intention to voluntarily disclose information about one’s (health) status to others [55]. The complete theoretical model is depicted in Figure 1.

### Methodology

We described the methodology along the Consolidated Criteria for Reporting Qualitative Research (COREQ) domains (Multimedia Appendix 2) [56].

#### Domain 1: Research Team and Reflexivity

##### Personal Characteristics

Interviews were conducted by 3 different interviewers who were part of the joint research project funding this research. All researchers had postgraduate degrees, while 1 also had a PhD; 1 of the researchers was female, while 2 were male. All interviewers had received prior training in conducting qualitative interviews, and all were employed at a research institution or university when conducting the interviews.

##### Relationship With Participants

The researchers conducting the interviews had no previous relationship with the interviewees. The participants received a 1-page introduction of the research project and its goals before agreeing to take part in the interviews. They did not have any further knowledge of the researchers other than project involvement. The researchers were involved in a common research project with the objective of developing a virtual consent assistant for informed and sovereign patient consent.

#### Domain 2: Study Design

##### Theoretical Framework

The study was based on a qualitative content analysis and followed a deductive category application [57,58]. Due to the exploratory nature of our study for the German system, we performed qualitative, semistructured interviews [59] to provide a starting point for our empirical assessment.

##### Participant Selection

Due to the COVID19 pandemic and associated contact restrictions, we were unable to proceed with our initial plan to recruit a variety of participants onsite at a large German university clinic. We evaluated different data-gathering strategies for their viability. We eventually recruited targeted interview candidates using a combination of purposive and convenience sampling. We selected candidates who (1) had signed a consent form for a medical procedure in the past 6 months and (2) met the rough replication of the demographics of potential app users from the existing personal networks of the researchers. The participants were approached via phone calls. Overall, we approached 25 people, of which 20 (80%) agreed to be interviewed. All participants were offered a small financial compensation (€25, or US $27) for their time. One person asked for the compensation to be donated to a worthy cause.

##### Setting

The interviews were conducted in German language and over the phone, whereby the participants answered the phone in their own homes. We could not assess whether there was anyone else present with them. The researchers worked out of their own offices and were by themselves. Overall, we conducted 20 qualitative user interviews. Table 1 shows the details of the participants.

**Table 1:** Descriptive information about participants.

Interview/participant number	Sex	Age (years)	Chronic illness present
1	Female	41	No
2	Female	45	Yes
3	Male	26	No
4	Female	40	Yes
5	Female	26	No
6	Male	29	Yes
7	Female	44	No
8	Female	60	Yes
9	Female	43	No
10	Male	47	No
11	Male	81	Yes
12	Female	70	Yes
13	Male	34	Yes
14	Female	34	Yes
15	Male	83	No
16	Female	Not available	Yes
17	Female	27	Yes
18	Female	81	Yes
19	Female	81	Yes
20	Female	30	No

##### Data Collection

We developed a questionnaire for the semistructured interviews, focusing on experiences with PHI consent, digital and consent literacy, trust, and individual data-sharing preferences. The translated questionnaire can be found in Multimedia Appendix 3. The questionnaire was developed by the researchers conducting the interviews and discussed in the project consortium. As shown in the provided questionnaire, we added a vignette as a final question in order to elicit the participants’ intent on sharing PHI based on a specific situation in line with Barter and Renold [60]. Before the interview, the participants were asked to fill out a short questionnaire for demographic data. No repeat interviews were carried out. One interview had to be paused because the participant had to take a call, and was continued soon after. All interviews were audio-recorded, and the research team took limited field notes during the interviews. The average interview time was about 60 minutes.

Metathemes, as defined by Guest [61], presented themselves after coding about half the interviews, and we could assume data saturation after analyzing all 20 interviews. The transcripts were not returned to the participants for comment.

#### Domain 3: Analysis and Findings

##### Data Analysis

The interviews were transcribed using a transcription service via commissioned data processing following all stipulated information security measures. The transcripts were imported into MAXQDA [62] for coding. Coding was performed independently by 2 researchers with postgraduate degrees, 1 of whom had a PhD. We revised the coding agenda and coding rules before final coding and then compared results after final coding using Cohen κ. We reached a Cohen κ value of 0.88, indicating solid interrater agreement [63]. An overview of the constructs and definitions of the coding agenda, key illustrative quotations per code, and the number of statements coded per interview can be found in Multimedia Appendix 1.

##### Reporting

In this paper, we presented quotations to illustrate our findings. All interview quotations presented in the results were translated from German to English. The interviews were numbered for identification, and the position (denoted as “pos.” in the quotations) of each quotation in the transcript was marked accordingly. We presented major and minor themes in the results, and we adapted our initial model according to our exploratory findings.

### Ethical Considerations

We obtained a positive ethics vote from the University of Cologne (review number: 21-1271). The survey was conducted in accordance with the applicable provisions of the Data Protection Act (Art.9 para.2 letter b DSGVO). The interviewers are subject to the obligation of secrecy and are also bound to data secrecy.

Prior to conducting the interviews, the participants obtained information about study participation and a consent form. The signed consent forms are kept separately from the short questionnaire and interview results in the university clinic so that no connection can be made between the information in the short questionnaire and the consent forms. The interviews were recorded with the help of a recorder. The recorded interviews were transcribed and pseudonymized. They were processed in written and pseudonymized form only so that it is no longer possible to draw conclusions about the person or third parties. In contrast to the transcripts, the audio files created could not be sufficiently pseudonymized for technical reasons, which is why they were not further processed after the interviews. They will, however, be stored until the end of the project in October 2023 and then deleted.

The participants were thoroughly informed that their participation in the study is completely voluntary. This means that at any time and without giving reasons, they had the right to refuse to answer individual questions. They could also terminate participation in the study or withdraw their consent to participate at any time without incurring any disadvantages. In this case, all data collected up to that point (questionnaires, transcripts, audio recordings) were completely deleted. All data collected in the context of the interview study were treated confidentially, stored exclusively for scientific purposes, and used exclusively by the scientists in the project team.

## Results

### Participant Details

The average age of the participants was 48.5 (median 43.0) years. The youngest participant was 26 years old, and the oldest was 83 years old. About 70% (n=14) of the participants were women. About 80% (n=16) had an academic degree. The sample was skewed and may have overrepresented women with higher education. We did, however, postulate that the gained insights were relevant, given the articulated need to improve the understanding of female health perceptions and behaviors [64,65]. Of the 20 participants, 12 (60%; n=10, 83%, female) suffered from chronic illnesses and were more frequently in contact with health care institutions. Most participants dealt with 2-5 medical consent forms annually. In addition, 5 (42%) participants, who all reported 1 or more chronic illnesses, stated they would be confronted with 6-11 consent forms, indicating a multitude of interactions with the health care system. All participants said they use technology, mainly smartphones and laptops, for personal communication and information purposes. The most used features are search engines (n=20, 100%), email (n=19, 95%), online shopping (n=19, 95%), and online banking (n=18, 90%). Only 12 (60%) participants reported the usage of social media, and only 10 (83%) participants reported using online education formats. Furthermore, 12 (60%) participants reported their digital aptitude with a 3.5-4 score on a 5-point Likert scale ranging from little to no knowledge to expert knowledge. In contrast, participants over the age of 70 years reported their digital aptitude with an average score of 2.6.

### Conceptual Model Validation

To validate the conceptual model of eHealth trust formation in Figure 1, we analyzed our results along 3 stages: belief formation, attitude formation, and information-sharing intent. Generally, we found that most people show a behavioral intent to share their PHI with health care professionals digitally. One participant stated:

I am 100% convinced that the pros outweigh the cons.

Interview 2, pos. 121

Another stated:

If you can judge the risk [associated with data breaches when sharing], then, generally yes, I would share it.

Interview 20, pos. 201

Given the fulfillment of certain conditions, such as anonymity, participants would be willing to share their PHI with (noncommercial) medical research institutions for advancement in medical science. One participant stated:

And I think it is very important that everything that is related to [human health] is made available to science.

Interview 11, pos. 11

#### Stage 1: Belief Formation

##### Technology Experience

In the belief formation stage, we found that an individual’s privacy concern is indeed influenced by previous experiences with technology (tech-savviness) and their knowledge on information security. As previously mentioned, all participants used digital technology, mainly smartphones and laptops. The median self-reported tech-savviness was 3.5 on a 5-point Likert-Scale, with 1 being low and 5 being high. A notable statement was:

I would check the possibilities suggested on my computer or phone, and then I would check settings to see what I want and don’t want, and if I don’t understand it, then [the app] I would delete it.

Interview 16, pos. 77

People with low tech-savviness (mostly over the age of 70 years in our sample) adopt strategies to help interact with digital technology. This was indicated in this statement:

If I need a new app or want to delete one, then someone has to do this for me.

Interview 11, pos. 55

The overall knowledge on information security can be classified as low to medium and heavily relies on what has been communicated by the provider and preinstalled in the used system. One participant mentioned:

Something like this is already on my phone, an antivirus program.

Interview 10, pos. 67

Often, people do not seem to be aware of or interested in the subject, as indicated by, for example, participant 2 (pos. 53), who “never looked into it.” People seem to assume that:

As soon as you are digital or you are transferring information, then you can’t control where it ends up and who uses it.

Interview 12, pos. 71

Participants with a higher level of digital competency stated:

Not every app gets a right to access things where I don’t think the app needs them.

Interview 1, pos. 71

There is no security measure that cannot be hacked…Because otherwise you would not be able to operate it, if it was completely secure.

Interview 14, pos. 107

eHealth awareness does not seem to play a predominant role.

##### Policy and Regulation

Information security policies and the regulatory framework for data sharing pose another antecedent to privacy concerns. Participants statements included “if it is encrypted […] then I don’t see a problem” (interview 9, pos. 137), “always using the latest standard of anonymization […] and ensure transparency” (interview 14, pos. 171), and “so that no third party can access the data, but only the person that one has consented to” (interview 16, pos. 47). When sharing data for medical research, most participants wanted to stay completely anonymous. One participant, however, stated:

I would share my data with the condition that I get informed when they find something. That would be useful for me as a prophylactic measure.

Interview 12, pos. 145

This indicates that complete anonymity may not always be beneficial for the data owner. We analyzed data from a German health care system, implying strict regulation on information sharing and information usage, which aids participants in feeling secure when sharing PHI. A notable statement was made by a participant who is an immigrant:

If you are here in Germany and know that everything is checked and done meticulously, then I don’t have a problem [with sharing my data]. In [the country] where I am from, you don’t know what they do with the [data]. There, I would think twice about it.

Interview 2, pos. 17

Participants said they are comfortable sharing PHI within Germany or the European Union (EU) but are wary about sharing PHI with institutions outside the EU, as indicated, for example, by participant 16:

I would trust [institutions] within Europe.

Interview 16, pos. 173

##### Demographics

Considering demographics, age was found to be the most predominant factor influencing privacy concerns. Participants stated:

So if I was 75, then I would say, I don’t care, take my data. Because I think, ok, then hackers have my data, but what are they going to do with it? But not in my current age.

Interview 7, pos. 133

If you would ask someone who is 20, 30, or 40, they would give a different answer because everything happens digitally for them.

Interview 12, pos. 39

All other factors were barely found in the interviews.

##### Personality and Disposition

The final antecedent was personality and disposition. In our sample, we found evidence for the importance of disposition to trust when sharing PHI. Most participants exhibited a tendency to trust and mentioned that a base level of trust is needed in all social and digital interactions. This was indicated by statements such as:

You need to have a level of trust these days, both in technology and in relation to the digital possibilities we have today.

Interview 15, pos. 105

Then I have to give them the benefit of the doubt, that the information is important, and that’s what you need to have in general towards a doctor and a hospital.

Interview 3, pos. 23

One participant, however, stated:

This is difficult. I trust myself...I don’t trust anyone. This is based on my experience.

Interview 10, pos. 81

Participants were aware of the benefits of actively pursuing a healthy lifestyle (patient activation), and most stated that they try to do so, succeeding to a varying extent. Some participants mentioned a (brief) use of step counters or sleep trackers. They did not relate these statements to privacy concerns. We found evidence that showed an influence of patient activation on privacy concern formation, as indicated by the statement:

Yes, you see. Then we have a yes if I am affected myself.

Interview 10, pos. 149

Regarding the impact of health status on privacy concerns, we found ambivalent results. Some people with chronic illnesses were skeptical about sharing their PHI or believed it is not important, while others said they would happily share their PHI. There was no evidence that health concerns such as chronic illnesses have an influence on privacy concerns.

##### Privacy Concerns

All participants expressed some level of privacy concern. Participants had “the feeling, that my data already is everywhere anyway” (interview 5, pos. 51) and a feeling of “overstimulation due to too much information” (interview 17, pos. 105) and being unable to control it in the first place. This was fittingly expressed by participant 13:

Yes, because I always have this remaining risk that the data could be misused.

Interview 13, pos. 143

This was often mentioned in relation to a level of acceptance of the matter. Most participants stated the risk of “sensitive data in the wrong hands” (interview 3, pos. 151) through data leaks or hacking. Some worried about leaking illness-related PHI to employers and the resulting discrimination due to health concerns, as mentioned by participant 7:

If someone has an illness and she applies somewhere, [then] the potential employer could find that [the person] has an illness and not hire her.

Interview 7, pos. 13

Other participants mentioned unwanted targeted ads or discrimination.

##### Social Influence

In addition to privacy concerns, social influence also affects trust formation. Particularly, older participants actively rely on their children and grandchildren for support in IT and sharing decisions and involve them in their decision process. Participant 12, for example, stated:

Then I would ask the younger generation.

Interview 12, pos. 57

#### Stage 2: Attitude Formation

In the attitude formation stage, we differentiated between cognitive and emotional trust as well as the privacy calculus calculation.

##### Cognitive Trust

Overall, there was a high level of cognitive trust in medical institutions, such as hospitals, and other health care and health insurance providers:

You are willing to share your data as long as you trust the institution.

Interview 3, pos. 117

Because I have a base level of trust in our health care system.

Interview 8, pos. 21

We also found that a base level of trust is created through an in-person interaction with the treating physician or the health insurance provider, as indicated by participant 2:

If my family doctors said you need to monitor your blood pressure and I would like you to use this [app]…then I would use it.

Interview 2, pos. 79

However, trust in Big Pharma and the general intentions of companies using health-related data was low. Participants used large platforms, such as Facebook and Google, but tended to have reservations about their data collection and usage policies. One participant stated:

The motivation of the companies to get data is high…They surely get more information than they deserve.

Interview 1, pos. 89

This statement displays the influence of the person’s privacy concern on trust formation. Participants did, however, trust the expertise and integrity of, for example, the apps provided by their health insurance providers, as indicated by the following statements:

If you talk about expertise, they are all competent. Generally, I would say that everything related to health insurances and sport universities would have the highest level of expertise.

Interview 4, pos. 107

The health insurance app is competent because I can upload my bills.

Interview 6, pos. 151

##### Emotional Trust

Participants based their emotional trust in HIEs on their general disposition to trust and previous experiences expressed within privacy concerns, as indicated by this statement:

When the health insurance said we have this app and we would like you to use it…I thought I can do that for them…I have blind faith that the [health insurance] makes sure it is safe.

Interview 2, pos. 71

Depending on their dispositions and experiences, some participants showed an emotional mistrust in HIEs, such as 1 participant:

I would have a feeling, I don’t know, what types of data go where, what they can tell someone about me…I think this is too risky.

Interview 7, pos. 149

##### Privacy Calculus

In addition to forming a trust attitude at this stage, participants underwent a privacy calculus, comparing the utility and associated risks of sharing PHI. Participants were more likely to opt into sharing PHI if they perceived it to be beneficial (1) for their own convenience and usability of the app, (2) for their own health, or (3) if it aids a common good of advancing medical diagnosis and treatment. One participant stated clearly, “Yes, if I benefit from it,” (interview 5, pos. 123), while mentioning the common good, “So if I share this data for research purposes, then I definitely see the benefit that you can perform research with it. And that is somehow a priority for me” (interview 5, pos. 173).

Another one said:

[I’d] have a good feeling [that] things will be a bit easier…if the data is already saved.

Interview 8, pos. 117

… Because then you have your whole health history and all the relevant information in one spot…I believe this has a lot of potential.

Interview 8, pos. 41

Yet another participant stated:

I think it is very important that data is shared between the [medical] professionals.

Interview 11, pos. 23

Perceived risk is mainly associated with misuse of data by third parties, as indicated by participant 3:

…Sensitive data gets into the wrong hands…That would be bad for the user.

Interview 3, pos. 151

#### Stage 3: Intent Formation

In the information-sharing intent stage, participants formed their final intent toward sharing their PHI with an HIE. To assess intent, participants received a vignette (see Multimedia Appendix 1) and were asked for their recommendation. Most participants (n=19, 95%) displayed a positive intent to share their PHI, even given the special circumstances:

Because it will be beneficial for research on this illness […] I think you should do it, even if data is stolen.

Interview 6, pos. 227

##### Additional Themes

In addition to the deductive themes from the model, we found that participants preferred apps that are easy to use in daily life. Further, participants preferred a specific consent solution [66] compared to a broad consent solution.

**Figure 2:**
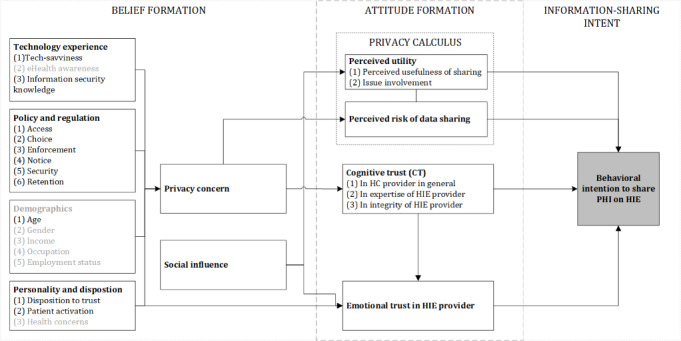
Updated trust formation model (own illustration). HC: health care; HIE: health information exchange; PHI: personal health information.

Figure 2 shows the updated eHealth trust formation model based on our interview results. The figure shows which constructs are supported by evidence within our data set and which ones are currently not supported. These results should certainly be validated with further qualitative and quantitative cross-country studies.

## Discussion

### Principal Findings

The objective of our study was to gain deeper insight into the issue of trust in HIEs and answer the following question: How does an individual form a behavioral intent to share PHI with an HIE platform? We contributed to the discussion of trust and informed consent in digital health in the following ways: First, we synthesized the main influence factors into a complex model of trust in HIEs. Next, we verified the influence factors through a qualitative analysis using patient interviews in the German health care setting. We showed which constructs are supported by evidence within our data set and which ones are not. Since this was an exploratory study, we did not adapt the model based on our current findings.

Our results showed that most patients generally have a positive attitude toward sharing their PHI digitally through an HIE. Our model provides a new point of view on the formation of a behavioral intention to share PHI by combining key concepts of the APCO model with a belief-attitude-intention framework and research on trust and the privacy calculus. Based on the interviews, we found that patients form a privacy concern in the belief formation stage based on antecedents, which can be divided into 4 categories: (1) demographics, (2) policy and regulation, (3) previous experiences with technology and information security, and (4) an individual’s own personality and disposition to trust. We also highlighted which factors appear more important in influencing the information-sharing intent. All participants in our sample use technology and gather their own experiences with it.

In the attitude formation stage, privacy concerns and social influence lead to the formation of trust in both cognitive and emotional terms. A base level of trust is created through in-person interactions with the treating physician or the health insurance provider. Trust is then transferred to the suggested HIE for sharing PHI. This is a crucial difference compared to trust formation in an e-commerce setting, for example, without contact with a physical party in the process. In the German health care setting, patients can choose their health care provider (within time and location restrictions). They can already develop a level of cognitive trust in the health care provider before interacting with the HIE. In addition to trust, the privacy calculus influences the intent to (not) share PHI with an HIE.

### Limitations

We based our model on previous empirical and theoretical research. Regarding the representativeness of our results, our sample was slightly skewed and may have overrepresented women with higher education. This may be due to increased digital health literacy [67,68] and an increased interest shown by this demographic in the topic [23]. The insights gained are relevant, given the articulated need to improve the understanding of female health perceptions and behaviors [64,65].

We did not interview people under the age of 25 years, which may have impacted the results. We did, however, capture some secondary insights into their attitudes through conversations with a younger age group mentioned by the participants. All participants displayed some level of tech-savviness, which may be due to low interest of non-tech-savvy people in the research and an unwillingness to participate.

The study did not capture real-life PHI-sharing decisions but, rather, analyzed the behavioral intent. Participants may have provided socially desirable answers, which may not be in line with their final action of sharing PHI. We did, however, assume that a positive intent will eventually lead to a positive action, that is, sharing PHI for the majority of participants [69].

The validation was conducted based on the results of 20 interviews with patients in Germany. To generalize the results, further qualitative and quantitative cross-country validations of the model are needed.

### Comparison With Prior Work

Our model provides a new point of view on the formation of a behavioral intention to share PHI by combining key concepts of research on privacy, user acceptance, and trust. With this, we address calls for a more nuanced view on the patient perspectives concerning privacy and trust [42]. We collected data from a country that is a front-runner for approving digital treatment options with cost coverage through statutory health insurance but at the same time has a comparatively rather low level of trust and high level of technology skepticism [4].

With our data, we confirmed previous findings [46,68] that most patients generally have a positive attitude toward sharing their PHI digitally through an HIE, even in the German health care setting [23,70]. Our data indicate that age is a predictor of privacy concerns [4]. Older participants stated that they are happy to share their PHI, which is in line with previous findings [71]. This could relate to a lack of understanding of what the shared PHI could be used for, which is in line with studies on digital (health) literacy [5,72,73]. However, it could also reflect the need to share information in order to enable a better understanding of the information for oneself [71]. The middle-aged participants in our study exhibited a higher level of privacy concerns. Studies [45,74] have found that adolescents and young adults exhibit fewer privacy concerns, possibly due to a limited understanding of the consequences as well. Further studies are needed to better understand the impact of age on privacy concerns, particularly for the older generation.

Our results further indicated that knowledge of and previous experiences with information security and technology might play an ambivalent role in forming privacy concerns: A higher level of knowledge could, on the one hand, decrease privacy concerns, as the individual knows which measures to take to mitigate the risk of a data breach. On the other hand, it may increase the level of privacy concerns, as the individual understands how easily data breaches can occur, even with measures in place. The latter is in line with the findings of Baruh et al [75].

In addition, a base level of trust is created through in-person interaction with the treating physician or the health insurance provider, which is in line with previous studies [8,31,50,76] and poses a stark difference to non-health-related information sharing, where there is rarely an in-person interaction required.

### Conclusion

Sharing PHI through or with HIEs has the potential to significantly improve the quality of care, patient outcomes, and satisfaction and to raise efficiencies in the health care sector. Privacy concerns and trust formation are a main pillar of successful and patient-centered introduction and usage of HIEs and EHRs. In terms of the practical implications of our study, patients generally have a high level of trust toward medical institutions and tend to be willing to share their PHI, given the fulfillment of certain antecedent conditions by HIEs providers, such as information security, risk mitigation, transparency, anonymity, and a defined group of (noncommercial) users.

Offering educational measures as well as the option for specific consent [66] may increase patients’ trust and their intention to share PHI. Increasing patients’ knowledge appears essential in facilitating empowerment and awareness of data sovereignty, despite the potential effect on privacy concerns. Developers of HIE solutions should, along the lines of General Data Protection Regulation (GDPR) requirements, aim to educate users (both medical and nonmedical staff as well as patients) on the implications of their choices. They should enable patients to choose sharing options based on their personal knowledge and preferences. Arguably, however, implementing privacy by design and security by design in old implementations of systems proves to be more difficult compared to new applications.

HIE providers need to clearly communicate the benefits of their solutions and information security measures to both health care providers (physicians, nursing and administrative staff) and patients in terms of convenience, health benefits, and public welfare. Health care providers are key partners of HIE developers with regard to sharing PHI. This entails that creating trusting relationships with physicians and health care staff, as well as national health organizations, is essential to increase patients’ PHI sharing. Medical professionals need to be convinced that the technology provides benefits, not only for the patient and related care activities, but also for internal service provision processes entailing time and cost savings for the practitioners. Implementing digital services must facilitate care delivery rather than producing additional work for the care provider. HIE developers should integrate care providers into their service development to better adapt their product to user needs. Another strategy may be to aim for a national rollout through a governmental organization to create a base level of trust.

In terms of usability, HIE providers should aim at making it easy for health care providers and patients to access, use, and navigate their apps. This could be done by, for example, performing early usability testing and offering access through multiple operating systems. Offering (non)monetary compensation for sharing certain types of PHI with commercial parties could create an additional incentive for partaking in commercial research, which is needed to bring medication and treatments to market.

## Appendix

Multimedia Appendix 1 Overview of constructs, definitions, illustrative quotes, and frequencies.

Multimedia Appendix 2 Consolidated Criteria for Reporting Qualitative Research (COREQ) checklist.

Multimedia Appendix 3 Questionaire (translated).

## Abbreviations

APCO: Antecedent-Privacy Concern-Outcome
EHR: electronic health record
HIE: health information exchange
mHealth: mobile health
PHI: personal health information
UTAUT: Unified Theory of Acceptance and Use of Technology
UTAUT2: Extended Unified Theory of Acceptance and Use of Technology
