# Supplementary material for: Trust and Health Information Exchanges: Qualitative Analysis of the Intent to Share Personal Health Information
Source: J Med Internet Res. 2023 Aug 30;25:e41635. doi: 10.2196/41635 (PMC10500360; doi:10.2196/41635)
Supplement: Multimedia Appendix 3 [file jmir_v25i1e41635_app3.docx]

**Interview guide – translated into English**

| **Item** | | | | | | | **Interview text** |  |
| --- | --- | --- | --- | --- | --- | --- | --- | --- |
| Personal introduction [this part can be completely customized] | | | | | | | Thank you for taking the time to partake in this interview today. My name is [name] and I work as [job title] at [institution] and would like to interview you today as part of the [name] project. |  |
| Explanation of the research project | | | | | | | Before we begin, I would like to say a few words about the [name] research project.  The aim of our research project is to develop a new digital tool that enables patients in hospitals and doctors' offices, as well as participants in medical research, to make informed and self-determined decisions about who can access which of their personal health information and when. Until now, consent has been largely paper-based. Moving into the digital space holds many potential benefits but also dangers. For this reason, I am very pleased that you are supporting me and my colleagues in creating a basis of trust for digital consent with this interview today. |  |
| Data protection information and consent | | | | | | | I will now switch on the recording device for our interview and then summarize once again the framework conditions of this interview, which you have already received in writing with the consent form.  [Switching on recording device]  At the beginning we ask you once again to tell us your code.  Now that you have received an overview of the content of the interview, I would like to ask for your permission to record the interview using a recording device. The interview is expected to last one hour. The recorded interview will be transcribed and pseudonymized so that no conclusions can be drawn about you as a person or about other persons. The data will be used exclusively within the framework of the [name] project and will be deleted after the legal retention period has expired. During the interview, you have the right to terminate the interview and withdraw your consent at any time without giving reasons. If you agree to this, please let me know now. If you have any open questions or comments about the consent, please feel free to express them now. |  |
| 1.0.a | | | | Introduction to the topic of informed consent in the medical treatment context | | | Please imagine that you are about to undergo a scheduled procedure at the hospital. While you are waiting at the hospital for this procedure, you will be asked to sign several consent forms for the processing of your personal health information. With these consents, you authorize the hospital to store your data in the in-house system, share your data with other stakeholders, and send medical samples to an external laboratory for testing. |  |
| **#** | | | | | **Item** | | **Interview text** |  |
| 1.1 | | | | Personal experience in the treatment context | | | What is your experience with consenting to the processing of your personal health information?  *Supplementary questions if necessary:*  *How often and to what extent have you had contact with providing consent?*  *To what extent have you informed yourself about consenting to the processing of your personal health information?* |  |
| 1.1.a | | | |  |  |  | **If intensive,** what experiences do you base this (self-assessment of your experience with consent processes as intensive) on? |  |
| 1.1.b | | | |  |  |  | **If weak, not at all**: What was the reason for this (the limited contact with consent processes in the past)? |  |
| 1.2 | | | |  |  |  | Do you still know exactly what personal health information was involved and for what purpose the data was to be collected and processed? |  |
| 1.0.b | | | | Introduction to the topic of informed consent in the medical research context | | | Now please imagine that the doctor treating you tells you that there is a remnant of the tissue samples that were sent to the laboratory for your treatment and this remnant could be used in medical research. In addition, the personal health information accumulated during your hospital stay could also help improve the organizational structure of hospitals. |  |
| 1.3 | | | | Personal experience in the research context | | | Have you ever made your data available for medical research? Please tell us about it. |  |
| 1.3.a | | | |  | | | **If so:**  What motivated you to share your personal health information?  Do you think there are good reasons not to make one's personal health information available to medical research? |  |
| 1.3.b | | | |  |  |  | **If no:**  Do you think it's important for people to support medical research with their health data?  In your opinion, are there good reasons not to make one's personal health information available to medical research? |  |
|  | | | | Transition | | | Now I'll move on to the next topic area, which will be about appropriate information for consent, information needs, and personal assessment of one’s digital literacy. |  |
| 2.1 | | | | | Subjective assessment of the need for information | | If you are asked to release your personal health information for medical treatment, what information do you personally consider important?  If you imagine your personal health information being used in medical research, is there additional information of interest to you in this context?  Have you ever educated yourself on issues related to consenting to the processing of your personal information? |  |
| **#** | | | | | **Item** | | **Interview text** |  |
| 2.1.a | | | | |  | | **If yes:** What topics have you been interested in this context? How did you inform yourself and which channels did you use to find information on the process?  *If needed analog, newspaper, brochure, personal conversation /digital "Google search", on websites, online videos, via email - own addition.* |  |
| 2.1.b | | | | |  |  | **If no:** Why not?  How do you generally go about finding out about a new topic?  Through which channels do you prefer to obtain information?  *If needed analog, newspaper, brochure, personal conversation /digital "Google search", on websites, online videos, via email - own addition.* |  |
| 2.2 | | | | | Preferred way to receive information | | How would you like to receive information in the event of an upcoming consent?  *Offer and ask when needed: via specific channels: analog, flyer, face-to-face/ digital email, app, info texts on websites, newsletter - own addition.*  *Presented in what way?*  *Exemplary preferred representations: Infotexts, infographics, videos - own supplementation* |  |
| 2.3 | | | | | Information time | | Is there a specific time when you would like to receive information about an upcoming consent for medical treatment or participation in a medical study?  *Supplement as needed: If you could choose, which situation is ideal for you to get information?*  *Are there also times or situations when you do not want to be informed?*  *Are there times or situations when the information process would disrupt you or when you do not have the capacity to absorb the information?* |  |
| 2.4 | | | | | Digital competencies regarding use, access, etc. | | Which digital devices do you use every day? |  |
| 2.4.a | | | | |  |  | How well do you think you can operate these devices? |  |
| 2.4.b | | | | |  |  | To what extent do you inform yourself about digital devices, even if you have no intention to buy? |  |
| 2.4.c | | | | |  |  | To what extent have you ever installed apps on the devices you use on your own?  *Supplement as needed: Did you have difficulties with this? Did you get help?* |  |
| 2.5 | | | | | Digital competencies regarding digital security | | To what extent can you check and change your privacy settings and app permissions on your smartphone?  What programs or settings do you use to protect your private information on your digital devices? |  |
| 2.6 | | | | |  | | Have you ever consented to the processing of your personal data online? |  |
| 2.7 | | | | |  | | Imagine you are using a new website, for example, that you don't understand at first glance. How do you learn to deal with it?  In what way do you deal with it? Independently? With the support of another person? |  |
| **#** | | **Item** | | | | | **Interview text** |  |
| 3.1 | | Introductory questions | | | | | What is trust for you? |  |
| 3.2 | | Attitude toward apps / Trust in apps / Willingness to use the app / Willingness to share data | | | | | Now think of an app or website you use that you find **particularly** trustworthy:   - - Which [app/website] are you thinking of right now?   - Why do you trust this [app/website]? What feature(s) does it have?   - Why is your trust in this [app/website] particularly high? |  |
| 3.3 | |  |  |  |  |  | Think of an app or website you use that you find untrustworthy:   - - Which [app/website] are you thinking of right now?   - Why do you trust this [app/website]? What feature(s) does it have?   - Why is your trust in this [app/website] particularly low? |  |
| 3.4 | |  |  |  |  |  | As briefly described above, many health apps offer you the opportunity to get information about health-related topics.  Would you use such apps in principle?  Why? |  |
| 3.5 | |  |  |  |  |  | Imagine you could use an app to give informed consent for the processing of your personal health information for medical purposes.  What [factors/criteria] would have to be met for you to trust this program? |  |
| ***3.6*** | | ***Introductory words to the validation block of the trust model*** | | | | | ***From a scientific perspective, the following points are also relevant for trust development in apps. Let us now turn to the first aspect*** |  |
| 3.7 | | Social inclusion | | | | | Now visualize persons who are important to you (family, friends).  What do you think these people think about such apps and how trustworthy do these people find these apps?  What advice would these individuals give you in dealing with an electronic consent form?  ***[Have it answered as an open question!]*** |  |
| 3.8 | | Concerns about your privacy | | | | | To what extent are you concerned that your personal  health information could be misused?  What could happen?  ***[Have it answered as an open question!]***  *If necessary, give example scenarios: a) Your illnesses/psychological disorders become known b) Miscarriages become known c) Hackers capture your data and blackmail you* |  |
| 3.9 | | Prevention | | | | | Do you believe that you can positively influence your health by following certain behaviors/habits?  If so, what behaviors would those be?  ***[Have it answered as an open question!]*** |  |
| 3.10 | | Competence attribution to the provider | | | | | Which health apps or websites providers do you know?  Which of them do you consider competent?  What do you base your assessment on?  ***[Have it answered as an open question!]*** |  |
| **#** | | **Item** | | | | | **Interview text** |  |
| 3.11 | | Integrity expectation to the provider | | | | | Which of these providers do you believe is managing your data the way he or she promises?  What do you base that on?  ***[Have it answered as an open question!]*** |  |
| 3.12 | | Emotional trust | | | | | How do you feel when you think about sharing your personal health information on health apps?  Why do you feel that way?  Does trust play a role here for you?  If so, in what form?  ***[Have it answered as an open question!]*** |  |
| 3.13 | | Weighing advantages anddisadvantages | | | | | Overall, when weighing the pros and cons of sharing your personal health information, do you believe it is too risky to share your personal health information, or do you believe the benefits of sharing personal health information electronically via an app/website outweigh the disadvantages.  What advantages and disadvantages do you see?  ***[Have it answered as an open question!]*** |  |
| 4.1 | | | Preferences regarding research objectives | | | | The following questions apply only to consents for the processing of personal health information for medical research |  |
| 4.1a | | |  |  |  |  | Are there any areas of medical research that you personally dislike?  Which ones?  *Examples if needed: Research to cure and prevent cancer, research to improve surgical practices in cosmetic surgery, research to optimize hospital economics, stem cell research, research to optimize genetics, research to assist patients at the end of life, research on abortion, research to improve anesthesia.* |  |
| 4.2b | | |  |  |  |  | Are there medical research goals that are particularly important to you?  Would you consent to processing your personal health information for this research? |  |
| 4.3a | | | Preferences regarding medical data types | | | | Is there any personal health information that you would make available to medical research without hesitation?  Which personal health information would that be?  *Examples, if needed: X-rays, patient record, blood sample analysis, tissue analysis, semen analysis.* |  |
| 4.3b | | |  |  |  |  | Is there any health data that you would not make available to medical research?  *Inquire if needed:*  *Which data are we talking about?*  *Why would you not want to consent to processing of this information?*  *How is this different from the personal health information you would share?* |  |
| **#** | | **Item** | | | | | **Interview text** |  |
| 4.4 | | | Preferences for non-medical data | | | | Is there any personal, non-medical information that you would make available to medical research?  Which ones and why would you consent to processing this information for research?  *Examples if needed: Data that your smartphone or smartwatch collects or the data you generate on social media. This can be GPS data, temperature, Facebook messages and much more.* |  |
| 4.5 | | | Preferences regarding data security | | | | Is there a certain security standard you would insist on when making your data available for medical research?  *Examples, if needed: Pseudonymization, encryption...* |  |
| 4.6 | | | Preferences regarding the research actors | | | | Are there specific research institutions or researchers that you would like to exclude from accessing your personal health information?  Examples if needed: a specific company, a pharmaceutical group, a research institution, etc. |  |
| 4.7 | | | Preferences regarding research funding | | | | Suppose researchers ask you if they can use your personal health information for a medical study. Suppose you want to make your personal health information available to a research project. To what extent might the funding type of the research affect the decision to release your data?  *Follow up if necessary:*  *Is there a type of financing that you prefer?*  *Is there a funding type that the reject?*  *Examples include: government funding, funding from a pharmaceutical company, funding from Google, funding from a social research fund.* |  |
| 4.8 | | | Preferences regarding the frequency of data access | | | | With every visit to the doctor and every use of a medical device, you continually produce personal health information. In your view, does it matter whether a research project processes this data once or whether your data is viewed at regular intervals over a period of several years? |  |
| 4.9 | | | Preferences regarding the transfer of data abroad | | | | Would you be willing to release your personal health information for a research project that is not conducted in Germany?  Are there countries for which you would not agree to a data transfer? |  |
| 4.10 | | | Thought experiment (vignette) | | | | Please imagine the following scenario:  Patient L has been very tired in the last couple of weeks and is not able to withstand stress. Her doctor sends her to the neurology department of a nearby hospital, where the doctors draw blood from her. The analysis of the sample indicated that Patient L suffers from a rare, incurable disease. The doctors ask Patient L to make her sample and other medical data available for research. As the disease is rare, the sample and data would contribute to advances and research and potential ways for treatment and cure. They inform Patient L that her health status will most likely not benefit from the research. Patient L is uncertain what to do, as there were recent news reports on data being stolen from a server with medical research data. The news stated that particularly patients with rare diseases would be easily identifiable within the stolen dataset. Do you think Patient L should make her data available for research? Why (not)? |  |
| **#** | **Item** | | | | | **Interview text** | | |
|  |  | | | | | We have now reached the end of our interview and would like to take stock together with you. | | |
|  | Benefits and disadvantages | | | | | Imagine you would use a digital consent assistant?  What benefits do you see in this?  What disadvantages do you see? | | |
|  | Expectations | | | | | What are your expectations of our project and the use of our digital consent assistant? | | |
| 5.1 | Conclusion | | | | | This brings us to the end of the interview.  Do you have any notes, comments, or questions for us?  Then thank you very much on behalf of the entire [project name] team for your efforts.  ***[Turn off recording device]***  Thank you again for your participation and goodbye. | | |
